# Epidemiological Insights into Feline Leukemia Virus Infections in an Urban Cat (*Felis catus*) Population from Brazil

**DOI:** 10.3390/ani14071051

**Published:** 2024-03-29

**Authors:** Laura Pancich Diesel, Lauren Santos de Mello, Weslei de Oliveira Santana, Nilo Ikuta, André Salvador Kazantzi Fonseca, Diéssy Kipper, Raquel Redaelli, Vagner Reinaldo Zingali Bueno Pereira, André Felipe Streck, Vagner Ricardo Lunge

**Affiliations:** 1 Veterinary Medicine Diagnostic Laboratory (LDMV), Institute of Biotechnology (IB), Postgraduate Programs in Animal Health (PPGSA) and Biotechnology (PPGBIO), University of Caxias do Sul (UCS), Caxias do Sul 95070-560, RS, Brazil; lauraphdiesel@gmail.com (L.P.D.); wosantana@ucs.br (W.d.O.S.); vagner20005@yahoo.com.br (V.R.Z.B.P.); afstreck@ucs.br (A.F.S.); 2 Simbios Biotechnologia, Cachoeirinha 94950-000, RS, Brazil; laurensmello@gmail.com (L.S.d.M.); ikuta@simbios.com.br (N.I.); fonseca@simbios.com.br (A.S.K.F.); diessykipper@hotmail.com (D.K.); 3 Gatices Veterinary Hospital, Caxias do Sul 95000-000, RS, Brazil; rredaelli@ucs.br

**Keywords:** FeLV, prevalence, Brazil, PCR, proviral load, viral load

## Abstract

**Simple Summary:**

Feline leukemia virus (FeLV) is spread through domestic cats (*Felis catus*) and has been associated with a wide spectrum of diseases, mainly leukemia and lymphoma. FeLV is highly prevalent in Brazil and other South American countries. The survival rate of cats persistently infected with FeLV is low, and many animals die within three years of infection. The present study aimed to investigate the epidemiology of this virus and related diseases in an urban cat population from Brazil. It was carried out with a total of 366 domestic cats in veterinary facilities from Caxias do Sul, a city in South Brazil. The results demonstrate that 109 (around 30%) cats were infected with FeLV and presented different disease outcomes (highlighting progressive and regressive outcomes). The main risk factors for cats becoming infected were the lack of a specific vaccination against FeLV and outdoor access. FeLV infection was also associated with apathetic behavior, lymphoma, and anemia. The present study provides important epidemiological insights into the FeLV domestic cat infections in Brazil, highlighting the high prevalence of the disease and its concerning clinical outcomes as well as the benefits of vaccination for the health of cats.

**Abstract:**

Feline leukemia virus (FeLV) is a retrovirus distributed worldwide in domestic cats and with different outcomes (progressive, regressive, abortive, focal). The present study reports an epidemiological survey of FeLV frequency and the evaluation of some risk factors and the two main disease outcomes (progressive and regressive) in an urban cat population from Brazil. A total of 366 cats with sociodemographic information and p27 FeLV antigen test performed were included in the study. FeLV DNA (provirus) in the blood samples of all cats was detected via real-time polymerase chain reaction (qPCR). Plasma samples from 109 FeLV-positive and FeLV-negative cats were also submitted to reverse transcription (RT-qPCR) to determine the FeLV viral load. The results demonstrated that 112 (30.6%) cats were positive through the p27 antigen and/or qPCR. A risk factor analysis demonstrated that cats without vaccination against FeLV (OR 9.9, *p* < 0.001), clinically ill (OR 2.9, *p* < 0.001), with outdoors access (OR 2.7, *p* < 0.001), and exhibiting apathetic behavior (OR 3.1, *p* < 0.001) were more likely to be infected with FeLV. FeLV-infected cats were also more likely to present with anemia (OR 13, *p* < 0.001) and lymphoma (OR 13.7, *p* = 0.001). A comparative analysis of the different detection methods in a subset of 109 animals confirmed FeLV infection in 58 cats, including 38 (65.5%) with progressive, 16 (27.6%) with regressive, and 4 (6.9%) with probably focal outcome diseases. In conclusion, this study demonstrates a high prevalence of FeLV in this urban cat population from Brazil and highlights the need to establish more effective prevention strategies (such as viral testing, vaccination programs, specific care for FeLV-positive cats) to reduce diseases associated with this virus in Brazil.

## 1. Introduction

Feline leukemia virus (FeLV) is a retrovirus that infects domestic cats (*Felis catus*) and is distributed worldwide. FeLV belongs to the family *Retroviridae*, subfamily *Orthoretrovirinae*, and genus *Gammaretrovirus*. It is an enveloped virus with single-stranded RNA [1]. FeLV replication requires proviral integration into the host’s DNA [2,3].

FeLV transmission mainly occurs through close contact between infected and naïve cats, usually through social interactions, such as mutual grooming and sharing food and water bowls [4]. Generally, an increased risk of FeLV infection typically includes young, unneutered males with outdoor access and living in multi-cat households [5].

FeLV infection can have different outcomes, including progressive, regressive, abortive, and focal (rare) infections [3]. With a progressive infection, infected cats become persistently viremic and can present many diseases (e.g., leukemia, lymphoma, etc.). In a regressive infection, FeLV replication is reduced with the effective immune response, despite provirus DNA being integrated into the host genome. With an abortive infection, animals spontaneously clear FeLV without the integration of the provirus into their genomes [3,4]. Therefore, FeLV-related diseases are usually detected in progressive cats, which exhibit a shorter lifespan than cats exhibiting the other outcomes [6]. Focal infections occur rarely and are characterized by persistent atypical local viral replication in tissues such as the mammary, salivary, and urinary epithelium. This focal viral replication can lead to discordant results from diagnostic methods [3,4].

FeLV diagnosis is usually performed through point-of-care enzyme-linked immunosorbent assay (ELISA) testing, which detects the p27 FeLV antigen. Polymerase chain reaction (PCR) and reverse-transcription (RT)-PCR are important diagnostic tools used to detect and quantify proviral DNA and viral RNA, respectively. The use of all these methods together has helped to differentiate progressive, regressive, and other infection courses [7,8].

Successful control programs have been implemented to remove FeLV-positive animals from the environment and prevent contact with healthy cats [9]. Individual testing and the isolation of positive cats, along with the vaccination of negative cats, are the most recommended preventive measures [3].

FeLV prevalence data varies from 2.4% to 3.6% in countries in the Northern Hemisphere, such as the United States, Canada, and Germany [10,11,12]. On the contrary, a much higher prevalence of FeLV infection (usually >20%) has been observed in different Brazilian regions according to recent reports [13,14,15,16]. The present study aimed to investigate the FeLV frequency, to identify the risk factors associated with FeLV infections, and to determine the main disease outcomes in an urban cat population from South Brazil.

## 2. Materials and Methods

### 2.1. Population, Data, and Sample Collection

**Figure 1:**
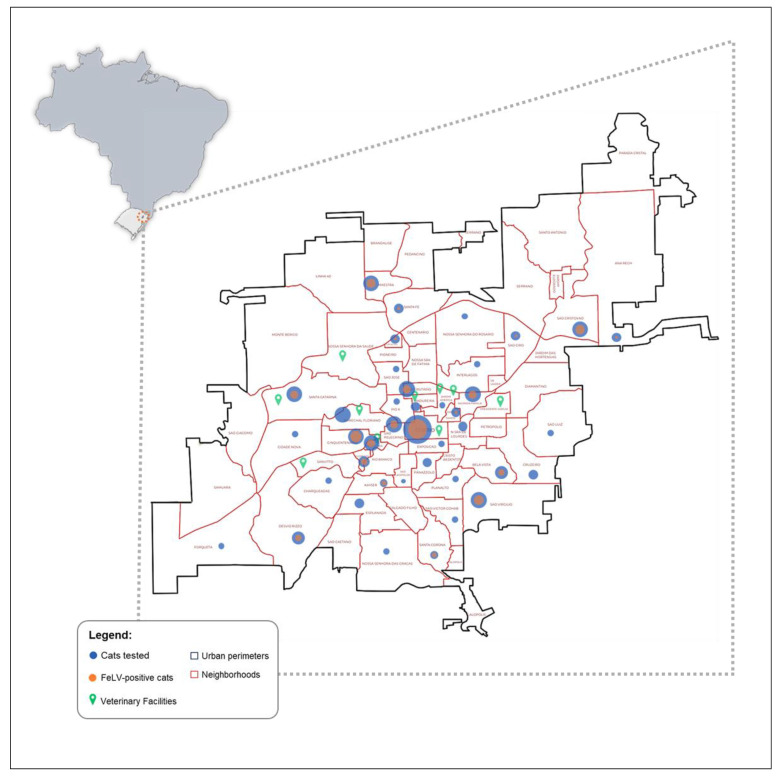
Map of Caxias do Sul, Rio Grande do Sul, Brazil, with the respective veterinary facilities as well as positive and negative cats in each neighborhood.

This study included a convenient sampling of privately owned cats attending nine different veterinary facilities in Caxias do Sul (Rio Grande do Sul state, South Brazil) from March 2021 to March 2023. This city is an important metropolitan center in South Brazil, and all samples were obtained from cats residing in more than 20 different neighborhoods in the urban area of the city (Figure 1). The Animal Ethics Committee (CEUA) of the University of Caxias do Sul (UCS) approved the study under the number 006/2022.

The owners of the cats that were examined at the partner veterinary clinics in this period were invited to include their animals in the study. All cat owners that accepted to participate in the study answered a questionnaire with the following information: age (years), sex, reproductive status (intact or neutered), behavior (alert or apathetic), health status, clinical signs and/or diseases presented, vaccination status, and outdoor access (yes or no). The clinical information was used to determine the health status (clinically healthy or sick). The major clinical manifestations were sorted by biological systems for further analysis. Samples with missing data information were categorized as “not informed”. According to their age, animals were divided into four categories, based on the definitions of feline life stages from the American Association of Feline Practitioners: kitten (up to one year), young adult (1–6 years), mature adult (7–10 years), and senior (>10 years) [17]. Finally, the owner’s home address was also recorded.

Using cat-friendly approach techniques for physical restraint, blood was collected through a puncture of the cephalic or external jugular vein. Feline retrovirus screening was performed using an individual SNAP FIV/FeLV Combo^®^ Test (IDEXX Laboratories, Westbrook, ME, USA). Additionally, a 0.5 mL whole-blood sample was collected from each animal and placed in tubes containing EDTA (ethylenediamine tetra acetic acid) anticoagulant and kept refrigerated (2 to 8 °C) to transport to the laboratory. All samples were further centrifuged (12,000 rpm for 3 min) to separate the main layers of the blood components: red blood cells at the bottom, buffy coat (containing the various white blood cells and platelets) in the middle, and the blood plasma at the top of the tube. These different blood layers were removed with a micropipette to be stored in separate cryotubes in a freezer (−20 °C) for all other laboratory analyses.

### 2.2. DNA Extraction and qPCR Assays

DNA extraction from blood cells with EDTA anticoagulant was performed using the commercial kits NewGene^®^ Prep and PreAmp (Simbios Biotecnologia, Cachoeirinha, RS, Brazil), according to the manufacturer’s instructions. qPCR assays were carried out with the total extracted DNA using NewGene^®^ FeLVAmp-qPCR master mix (Simbios Biotecnologia, Cachoeirinha, RS, Brazil) for the detection and quantification of FeLV provirus. All PCR runs were performed in a Step One Plus™ Real-Time PCR System Thermal Cycler (Applied Biosystems, Norwalk, CT, USA) under the following conditions: denaturation at 95 °C for 3 min, 40 cycles of 95 °C for 15 s, and then annealing/extension at 60 °C for 60 s. PCR amplification curves for all samples were evaluated in comparison with FeLV-positive controls. Reaction tubes without any DNA template were also included as negative controls in all independent runs. Samples were considered positive when presenting a characteristic amplification curve with a cycle threshold (Ct) value below 38 (Ct < 38).

Quantitative data were obtained using custom synthetic nucleotides (gBlocks; IDT, Coralville, IA, USA), which had oligonucleotide sequences of FeLV targeted by the same specific primers used in the commercial assays described above. These synthetic DNAs were ten-fold diluted to have pre-defined amounts of specific gene targets in the PCR assays. Standard curves for each assay were generated based on the cycle threshold (Ct) values of the ten-fold serial dilution of the synthetic DNA results. All real-time PCR cycle threshold (Ct) values for FeLV DNAs were then used to quantitate the values of provirus loads. The linear range was examined by plotting the data and comparing them to a line of equality. FeLV provirus concentrations were presented in log10/mL.

### 2.3. RNA Extraction and RT-qPCR Assays

RNA extraction from blood plasma was performed using the same commercial kits described in the previous section and according to the manufacturer’s instructions. RT-qPCR assays were carried out with total extracted RNA using the NewGene^®^ FeLVAmp-RT-qPCR reagents (Simbios Biotecnologia, Cachoeirinha, RS, Brazil). All RT-qPCR runs were also performed in a Step One Plus™ Real-Time PCR System Thermal Cycler (Applied Biosystems, Norwalk, CT, USA) under the following conditions: reverse transcription at 37 °C for 30 min, denaturation at 95 °C for 3 min, 40 cycles of 95 °C for 15 s, and then annealing/extension at 60 °C for 60 s. Reaction tubes without any DNA template were included as negative controls in all independent runs. Viral load quantitative data were obtained using the same procedure described in the previous section (synthetic DNA, ten-fold diluted).

### 2.4. FeLV Infection Disease Outcomes

FeLV infection outcomes were divided into four categories according to p27 antigen testing and FeLV DNA analysis: (1) not infected (p27 antigen-negative, provirus-negative), (2) regressive (p27 antigen-negative, provirus-positive), (3) progressive (p27 antigen-positive, provirus-positive), (4) focal (p27 antigen-positive, provirus-negative) [3]. FeLV DNA and/or RNA positive samples were additionally classified according to the quantitative results into low (<10^3^ virions copies/mL) and high (10^3^ virions copies/mL) proviral and viral loads.

### 2.5. Statistical Analysis

All evaluated data were analyzed using IBM SPSS^®^ software, version 23.0 (Armonk, NY, USA), and R Studio (Boston, MA, USA). For continuous data, normality was assessed using the Kolmogorov–Smirnov test with Lilliefors correction. Non-parametric quantitative variables were presented as median, minimum, and maximum values, with *p*-values obtained using the Mann–Whitney U test. Bivariate analyses were conducted to assess the association between categorical variables and the outcome, as well as to obtain the crude odds ratio with its respective 95% confidence interval (CI 95%). Absolute and relative frequencies were estimated for categorical data using either the Pearson chi-square test or Fisher’s exact test, as appropriate. Variables with *p*-values < 0.20 in the bivariate analysis were included in the multivariate analysis. In the final multivariate model, performed using binary logistic regression, *p*-values < 0.05 were considered statistically significant.

## 3. Results

### 3.1. Population Characteristics

A total of 366 samples were obtained from seven animal care clinics and two veterinary hospitals in the city of Caxias do Sul. The total sampling population included 200 male (54.6%) and 166 female (45.4%) cats from 44 different neighborhoods in Caxias do Sul, Rio Grande do Sul, Brazil (Figure 1). Information regarding reproductive status was available for 363 cats (99.2%), with 250 (68.3%) spayed/castrated and 113 (30.9%) sexually intact animals. Age data were available for 359 (98.1%) cats, and 206 (56.3%) were young adults (1–6 years); 83 (22.7%), kittens; 36 (9.8%), mature adults; and 34 (9.3%), seniors. Approximately half of all cats had outdoor access (n = 187, 51.1%), while 168 (45.9%) did not (missing information for 11 animals, 3%). Most animals were also clinically sick (n = 202, 55.2%), while 159 (43.4%) cats were healthy at the time of veterinary consultation (health status was not recorded for 5 animals, 1.4%).

Finally, 185 (50.5%) cats never received any vaccination, while 172 (47%) had been vaccinated at least once (vaccination history was not recorded for 9 cats, 2.5%). Importantly, the type of vaccine administered (with or without FeLV protection) was available for only 97 cats. Of these, 62 (63.9%) were vaccinated against FeLV.

### 3.2. FeLV Frequency and Risk Factors

A total of 120 cats (30.6%) were positive for FeLV in the p27 antigen SNAP test and/or the qPCR. In the comparison of these two different detection methods, there were 302 (87.4%) concordant results. On the other hand, 24 (6.6%) samples presented positive in the p27 antigen test and negative in the qPCR, while the other 22 (6%) animals resulted negative in the p27 antigen test and positive in the qPCR.

In the analysis of the geographic distribution in the city of Caxias do Sul, there were FeLV-positive cats in 24 different neighborhoods, highlighting Centro (n = 29/79, 36.7%), Santa Catarina (n = 6/26, 23.1%), Cinquentenário (n = 9/20, 45%), Pioneiro (n = 7/19, 36.8%), and São Pelegrino (n = 7/19, 36.8%).

FeLV was frequent in males (n = 64, 32%) and females (n = 48, 29%) (OR 1.2, *p* = 0.524). Also, FeLV infection occurred in neutered cats (n = 80, 21%) and sexually intact cats (n = 32, 40%) (OR 0.8, *p* = 0.482). Regarding age, young adults (1–6 years) were affected more frequently (n = 63, 31%) than kittens (≤1 year; n = 23, 28%), mature adults (7–10 years; n = 15, 42%), and seniors (>10 years; n = 8, 23.5%). Being a mature adult or a senior cat was identified as a protective factor (OR 0.1, *p* < 0.001 and OR 0.1, *p* < 0.001, respectively). Clinically sick cats were more frequently FeLV-infected (n = 81, 40%) than clinically healthy cats (n = 30, 19%) (OR 2.9, *p* < 0.001). A higher prevalence of FeLV was observed among cats that had outdoor access (n = 76, 41%) than in those that remained exclusively indoors (n = 34, 20%) (OR 2.7, *p* < 0.001) (Table 1).

FeLV-positive cats were approximately three times more likely to be apathetic (*p* < 0.001) and sick (*p* < 0.001) than uninfected cats. FeLV-infected cats were also more likely to have lymphoma (OR 13.7, *p* < 001) and leukemia (OR 13, *p* < 0.001) (Table 2). Finally, cats without specific immunization against FeLV (unvaccinated or vaccinated with vaccines without FeLV antigens) were more frequently infected by this virus (OR, 9.9, *p* < 0.001) compared to vaccinated cats (receiving the “quintuple” vaccine, including the FeLV antigen).

**Figure 2:**
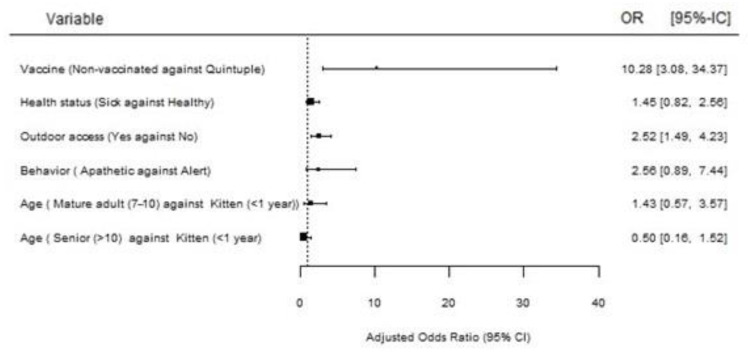
Factors associated with FeLV infections after multivariate analysis.

All risk factors associated with FeLV infection were compared in a multivariate analysis (Figure 2). Variables with statistically significant results were non-vaccinated (OR 10.3, 95% CI: 1.9–23.7) and outdoor access (OR 2.5, 95% CI: 1.5–4.2). 

### 3.3. FeLV Different Courses of Infection

A total of 109 cat blood samples (51 FeLV-negative and 58 FeLV-positive) could also have been quantitatively evaluated via qPCR and RT-qPCR to determine FeLV proviral DNA and viral RNA loads, respectively. According to the p27 antigen and FeLV DNA proviral results, these cats could be classified into four main possible diseases outcomes (Table 3).

All the 51 p27 antigen and FeLV proviral DNA negative samples were also FeLV viral RNA negative in the RT-qPCR, confirming that these cats were FeLV-uninfected. Among the remaining 58 cats, (i) all four animals with suggestive focal infection (p27 antigen positive and FeLV DNA-negative) resulted negative in the RT-qPCR, (ii) 13 out of the 16 cats with regressive infection presented FeLV viral RNA, and (iii) 34 out of 38 cats with progressive infection presented FeLV viral RNA.

**Figure 3:**
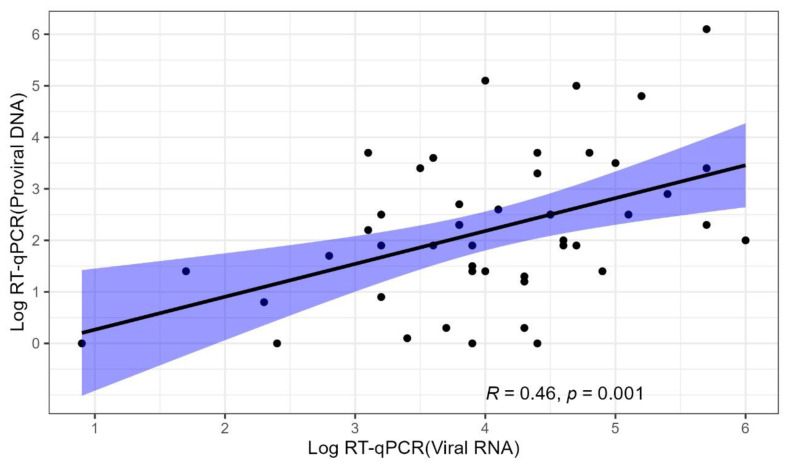
Scatter plot correlating FeLV proviral DNA and viral RNA loads.

Furthermore, FeLV DNA and FeLV RNA loads were also compared in a scatterplot (Figure 3). In most FeLV-positive cats, the DNA proviral loads were higher than 10^3^ and less than 10^6^ copies/mL, while the RNA viral loads presented values between 10^1^ and 10^4^ copies/mL. Importantly, cats with higher viral loads (>10^3^ copies/mL) also presented higher proviral loads (>10^3^ copies/mL), and this relationship was not proportional. In opposition, a wide variation in the FeLV proviral loads (from 10^1^ to 10^6^ copies/mL) was observed in cats with lower FeLV viral loads (<10^3^ copies/mL).

## 4. Discussion

FeLV is one of the main pathogenic and lethal viruses infecting domestic cats [4]. In Brazil, previous studies have reported high FeLV prevalence rates (from 26.9% to 31%), including in the South region [13,16]. The present study confirmed this FeLV high frequency since approximately 30% of the tested cats were FeLV positive in any testing procedure. This situation has resulted in many animal health concerns in the domestic cat populations, mainly due to the frequent transmission through close contact between susceptible and infected cats, the very high morbidity and mortality rates, and the absence of available specific antiviral treatments. In addition to this general epidemiological finding, the present study also provides additional data to understand the risk of FeLV infection in domestic cats from Brazil.

First, no significant association was observed between FeLV and sex or reproductive status, as previously published [13,18,19]. However, in FIV infection, a higher risk has been reported in non-neutered male cats [11,12,14], probably because the transmission of this other retrovirus occurs mainly through blood inoculation, is observed in fights, and is more frequently attributed to intact males. On the other hand, close friendly contact is the main route of FeLV transmission [4,20,21,22].

Second, the median age of cats infected with FeLV was two years, similar to that reported in other studies [11,23,24]. However, there was no significant difference in the median age of uninfected cats, which is probably related to the high prevalence, around 70%, of young cats under 4 years old in this study. Notably, old age (mature adults and seniors) was demonstrated to be a protective factor for FeLV infection. Factors potentially related to these aspects are the gradual decrease in susceptibility to FeLV with increasing age in addition to the shorter life expectancy in progressive cats [6,25,26,27].

Third, cats with outdoor access and in contact with other animals were more frequently infected with FeLV. This result was also expected according to previous studies demonstrating that FeLV frequency increases according to the number of contacts among cats [10,11,28].

Additionally, it was demonstrated that the risk of FeLV infection was 2.8 times higher in sick cats compared to healthy cats. Other studies have also shown that clinically ill cats were up to five times more likely to test positive for FeLV [9,17,29]. The prevalence of FeLV was close to 40% in studied cats with diseases, which is similar to that described in other places where the prevalence of this virus is also high [14,30]. FeLV-infected cats can be asymptomatic, but data the demonstrate that they frequently present with clinical manifestations [3].

A wide range of diseases such as neoplasms, bone marrow disorders, and immunosuppression may be associated with FeLV, mainly in cats with persistent viremia. Despite this, studies have shown that hematological disorders and lymphoma, the main neoplasia associated with FeLV, can also occur in non-productive infections [6,20,31,32]. Anemia is one of the most frequently described hematological disorders and clinical manifestations in FeLV-infected cats, as observed in the present study [6,13,24,33]. In addition, as reported by other studies, cats with anemia were more likely to be FeLV positive [24]. It is suggested that the occurrence of anemia is due to a specific FeLV subgroup, which interferes with essential mechanisms for erythropoiesis and leads to severe non-regenerative anemia and often causes death [20]. Anemia can also be a secondary alteration due to other infirmities, including neoplasia. Cats with FeLV infection have higher odds of presenting with neoplasia, especially lymphoma and leukemia. As in the studied cats, lymphoma is one of the most frequent diagnoses in infected cats, with a five-fold increased risk of cats with this neoplasm testing positive for FeLV being described [6,24,27,33,34].

The role of FeLV in oncogenesis may occur through mechanisms that include insertional mutagenesis, transduction, transactivation, and immunosuppression [35]. Lethargy is a non-specific clinical sign that was also more likely to be present in cats with FeLV. Its occurrence may be secondary to other alterations caused by FeLV, such as immunosuppression, which makes infected cats susceptible to other opportunistic pathogens [3,4,36].

Another very important contribution of this study is that it highlight the results of the relatively recent introduction of the practice of immunization against FeLV. It was demonstrated that unvaccinated cats were approximately 10 times more likely to be infected with FeLV than vaccinated cats, as also observed in another report [36]. Despite the lack of information on the type of vaccine used in the cats studied, there are currently several commercially available vaccines that may contain inactivated or recombinant viruses, highlighting the “quintuple” vaccine. Vaccination protects against the progressive form of the disease, although it does not necessarily prevent the integration of the provirus. The effectiveness of available vaccines is an aspect that is difficult to assess and may be linked to issues such as small study populations, support from manufacturers, and a lack of standard protocols for both testing and verification [3,37,38].

The determination of the proviral DNA and viral RNA loads in a subset of the total sampling population was also possible. These molecular assays have been very useful for monitoring FeLV infection as well as for determining the disease prognosis. Previous reports highlight that it is necessary to detect proviral DNA and viral RNA via PCR as well as through the p27 antigen using the ELISA test [4]. The comparative analysis of the FeLV DNA proviral and RNA viral loads of positive animals demonstrated a statistically significant positive correlation, that is, there was an increase in viremia according to higher amounts of FeLV proviral DNA. However, it is a weak association as only 21% of the variability could be statistically explained. Therefore, the remaining 79% of the variability is explained through other variables probably related to the different disease outcomes (progressive, regressive, and abortive infections).

The outcome of FeLV infection has generally been based on the results regarding p27 antigen and proviral DNA in most previous studies. Cats that test positive for p27 antigen and FeLV DNA provirus are classified as having a progressive infection, and those that are negative for p27 antigen and positive for FeLV DNA provirus are classified as having a regressive infection [4,15,39,40,41,42]. However, the use of only these viral detection methods has not allowed a complete assessment of the clinical picture due to the different possibilities of retroviral replication in the host as well as the several progressions and outcomes of FeLV diseases. The routine use of other laboratory analyses and retests have been recommended for more assertive clinical management [3,42]. In the present study, the progression of FeLV infection could be estimated using the qualitative results of the p27 antigen and proviral DNA, as well as FeLV DNA (proviral) and RNA (viral) load findings. Generally, cats with high FeLV DNA proviral loads also had elevated viral loads. Other studies have already demonstrated that cats with progressive infection have persistently high proviral DNA and viral RNA loads [6,37,43]. Progressively infected cats have been reported to have a median viral load of 4.7 × 10^7^ copies/mL plasma, with worse survival rates in cats with higher virus concentrations and antigenemia [37]. In the same study, it was described that cats with progressive infection had a plasma viral load greater than 10^3^ copies/mL since the initial week. However, this situation is different in cats with regressive infection. The FeLV viral load is reduced in these animals, and it was already demonstrated that most of the regressive cats had undetectable FeLV viral loads at the end of week 15 [37]. These different clinical conditions and FeLV disease progressions have resulted in a not strong association in the viral and proviral loads between progressive and regressive cats here and in other previous reports [6,7,44]. More studies are necessary to improve the use of laboratorial methods for the FeLV disease assessments in the feline clinical routine.

Regarding the outcome of FeLV infection, a higher proportion of cats was classified as progressively infected compared to regressively infected. This result is probably related to the high circulation of this virus reported in the southern region of Brazil and also due to the chosen urban cat population from veterinary clinics [13,14]. FeLV-related diseases are observed mainly in progressive infections, which may also have contributed to the detection of this type of infection in a cat population selected by convenience in veterinary clinics [9,12,29].

However, it is important to point out that the present work does not intend to propose criteria for determining the true outcome of the FeLV infection. It was also not possible to establish the definitive FeLV disease outcome in the evaluated cats because the testing in this study was performed on one unique sample from each animal. A more accurate diagnosis requires retesting the animals. In addition, this study is not definitive enough to affirm conclusively that FeLV is highly prevalent in this Brazilian region. It is important to note that the cat population was selected by convenience, including animals from veterinary facilities, many of them looking for clinical care due to different diseases [9,11,29,45]. The occurrence of discordant cases between tests (p27 antigen, FeLV DNA, and FeLV RNA) is also a situation that can occur in epidemiological studies with a large number of analyses, as observed in other studies [46,47,48]. These types of discordant samples may be due to false positive results in terms of the p27 antigen such as that described in cats with FeLV focal infections [42] or other hematopoietic disorders [47,48].

Finally, this study highlights that vaccination against FeLV was by far the most important preventive measure, with a 10-fold lower risk for FeLV infection. This epidemiological finding reinforces the importance of vaccination programs in preventing FeLV infection. The effective control of this virus also requires the widespread use of FeLV testing programs in Brazil.

## 5. Conclusions

The present study demonstrated a high prevalence (>30%) of FeLV in an urban cat population from Brazil, as well as the main clinical manifestations (anemia, lymphoma) and the concerning outcomes (with a high number of animals with progressive infection). It also highlights the benefits of vaccination against FeLV. It is necessary to establish more effective prevention strategies to reduce transmission and progressive infections in domestic cats from Brazil.

## Figures and Tables

**Table 1:** Risk factors associated with FeLV infections.

Variable	FeLV-Positive N	%	FeLV-Negative N	%	OR (95% CI)	*p*
**Age (years)**	2^a^ (0.16^b^–15^c^)	-	3^a^ (0.16^b^–18^c^)	-	-	0.665 ^d^
**Sex**						
Male	64	32.0	136	68.0	1.2 (0.4–1.8)	0.524^e^
Female	48	29.0	118	71.0	1.0 Ref.	
**Reproductive status**						
Intact	32	40.0	81	60.0	0.8 (0.2–1.4)	0.482^e^
Neutered	80	21.0	170	79.0	1.0 Ref.	
**Vaccine**						
Non-vaccinated	89	40.5	131	59.5	9.9 (3.5–28.1)	**<0.001^f^**
Vaccinated	4	6.5	58	93.5	1.0 Ref.	
**Health status**						
Sick	81	40.0	121	60.0	2.9 (1.8–4.7)	**<0.001^e^**
Healthy	30	19.0	129	81.0	1.0 Ref.	
**Outdoor access**						
Yes	76	41.0	111	59.0	2.7 (1.7–4.3)	**<0.001^e^**
No	34	20.0	134	80.0	1.0 Ref.	
**Behavior**						
Apathetic	32	52.5	29	47.5	3.1 (1.7–5.4)	**<0.001^e^**
Alert	80	26.5	222	73.5	1.0 Ref.	
**Age**						
Young adult (1–6 years)	63	31.0	143	69.0	1.1 (0.7–2.0)	0.629^e^
Mature adult (7–10 years)	15	42.0	21	58.0	0.1 (0.0–0.2)	**<0.001^e^**
Senior (>10 years)	8	23.5	26	76.5	0.1 (0.2–0.2)	**<0.001^e^**
Kitten (≤1 year)	23	28.0	60	72.0	1.0 Ref.	

^a^ Median; ^b^ Minimum; ^c^ Maximum; ^d^ Mann–Whitney’s U Test; ^e^ Chi-Square Test; ^f^ Fisher’s Exact OR: Odds Ratio. *p* ≤ 0.20 values are shown in bold.

**Table 2:** Major clinical problems presented by FeLV-infected and uninfected cats.

Major Clinical Problem	FeLV + (n = 112)	%	FeLV − (n = 254)	%	OR	*p*
Anemia	19	83.0	4	17.0	13 (4.2–38.5)	**<0.001^a^**
Non-specific clinical signs	12	54.5	10	45.5	2.9 (1.2–6.7)	**0.012^b^**
Other	12	50.0	12	50.0	2.4 (1.0–5.6)	**0.033^b^**
Lymphoma	12	84.6	2	15.4	13.7 (3.0–63.0)	**0.001^a^**
Renal disease	7	33.3	14	66.7	1.1 (0.4–2.9)	0.078^b^
Respiratory disease/signs	7	43.7	9	56.3	1.8 (0.7–5.0)	0.243^b^
Skin disease	7	46.7	8	53.3	2.0 (0.7–5.8)	0.168^b^
Mycoplasmosis	5	100.0	0	0.0	3.4 (2.9–4.0)	**0.003^a^**
Trauma	3	25.0	9	75.0	0.7 (0.2–2.8)	>0.999^a^
Gastrointestinal disease/signs	3	16.7	15	83.7	0.4 (0.1–1.5)	0.293^a^
Oral disease	3	50.0	3	50.0	2.3 (0.5–11.6)	0.375^a^
Neurological disorders	3	60.0	2	40.0	3.5 (0.6–21.0)	0.170^a^
Hepatobiliary disease	1	20.00	4	80.0	0.6 (0.1–5.1)	>0.999^a^
Endocrinopathies/metabolic diseases	1	16.0	5	83.0	0.4 (0.1–3.9)	0.671^a^
Leukemia	1	100.0	0	0.0	0.8 (0.0–18.8)	0.867^a^
Ocular disease	0	0.0	1	100.0	0.8 (0.0–18.6)	0.861^a^
Other neoplasms	0	0.0	5	100.0	0.2 (0.0–3.7)	0.280^a^

^a^ Fisher’s Exact Test; ^b^ Chi-Square Test; OR: Odds Ratio. *p* ≤ 0.20 values are shown in bold.

**Table 3:** The outcomes of FeLV exposure in 109 cats based on p27 antigen and FeLV proviral DNA data.

Outcome	p27 Antigen	FeLV DNA	No. of Cats/Total	% of Cats
FeLV-uninfected	Negative	Negative	51/109	46.8
Focal (suggestive)	Positive	Negative	4/109	3.7
Regressive	Negative	Positive	16/109	14.7
Progressive	Positive	Positive	38/109	34.8

## Data Availability

Data are contained within the article.
